# Advancements in Nanomaterial-Based Biosensors for Neuropsychiatric and Neurodegenerative Diagnostics: From Biomarker Discovery to Clinical Translation

**DOI:** 10.3390/bios16060327

**Published:** 2026-06-05

**Authors:** Xinyue Li, Xiaopeng Han, Qing Han, Xuan He, Yixin Huang, Aimei Liu

**Affiliations:** School of Life Sciences and Health, University of Health and Rehabilitation Sciences, Qingdao 266113, China; lixinyue@uhrs.edu.cn (X.L.); hanxiaopeng@uhrs.edu.cn (X.H.); hanqing@uhrs.edu.cn (Q.H.); hexuan@uhrs.edu.cn (X.H.); huangyixin@uhrs.edu.cn (Y.H.)

**Keywords:** nanobiosensors, neurodegenerative disorders, neuropsychiatric disorders, clinical translation

## Abstract

Nanobiosensors, with their unique physicochemical properties, are transformative tools for diagnosing and monitoring neurodegenerative diseases and mental disorders. This article systematically reviews the latest progress of nanomaterial systems and integrated sensing modalities in neurological disease diagnosis. First, we clarify the multiple functional roles of nanomaterials in biosensors, including signal amplification, interface optimization, and spatial positioning, and compare the applicable scenarios of various sensing principles based on different nanomaterials. Second, we evaluate the design and integration strategies of molecular recognition elements (antibodies, nucleic acid aptamers, molecularly imprinted polymers, and CRISPR-Cas systems) and discuss their synergistic integration mechanisms for improving detection performance. In terms of detection targets, we focus on three applications: high-sensitivity quantification of established protein biomarkers, real-time monitoring of dynamic neurochemicals (dopamine, serotonin, glutamate), and emerging liquid biopsy targets such as exosomal cargo and circulating microRNAs. Finally, to address the core challenges of biofouling, sensitivity–selectivity trade-offs, and multiplex detection in complex matrices, we propose three breakthrough directions for next-generation diagnostics: deep integration of multimodal and multiplexing platforms, closed-loop chemical brain–computer interfaces (cBCIs), and AI-driven predictive diagnostic models, collectively enabling a transition from passive detection to active sensing and intervention for precise, rapid, and non-invasive neurological disease management.

## 1. Introduction

Neurodegenerative diseases and mental disorders represent two major categories of neurological disorders, both of which have high incidence and prevalence rates worldwide. However, the diagnostic challenges they present are fundamentally different in nature. For neurodegenerative diseases such as Alzheimer’s disease (AD) and Parkinson’s disease (PD), the core diagnostic challenge lies in the issue of “lag.” These conditions progress slowly, with pathological changes, such as Aβ deposition and tau hyperphosphorylation in the brains of AD patients, occurring more than a decade before the onset of clinical symptoms [1,2,3,4]. Nevertheless, current clinical diagnoses are typically made only after overt symptoms emerge, at which point neural damage has often become irreversible [1]. Existing interventions offer only limited efficacy in the advanced stages of these diseases [1,5], making early diagnosis a critical factor in improving patient prognosis. In contrast, for mental disorders such as depression and schizophrenia, the primary diagnostic dilemma stems from “subjectivity.” These conditions lack objective and quantitative biochemical indicators, and diagnosis relies heavily on symptom-based assessments. The complex interplay between schizophrenia and affective symptoms further complicates differential diagnosis [6]. The absence of reliable biomarkers not only impedes precise disease classification but also restricts the development of personalized treatment strategies [6].

The current challenges in diagnosing neurological diseases largely stem from the inherent limitations of conventional modalities, such as ELISA, MRI/PET, and mass spectrometry (Table 1). Specifically, insufficient detection sensitivity often leads to delayed diagnoses, while the invasive nature of many procedures limits their clinical utility. For example, although cerebrospinal fluid (CSF) analysis reliably reflects central nervous system pathology, the highly invasive lumbar puncture required precludes its widespread use for routine screening and continuous dynamic monitoring [7]. Neuroimaging techniques such as MRI and PET are not only costly but also tend to yield positive results only when obvious structural or metabolic abnormalities have already developed during disease progression, making them inadequate for very early diagnosis [1,8]. In the field of biomarker detection, the issue of insufficient sensitivity is particularly acute: molecular events in the early stages of disease are associated with extremely low concentrations of biomarkers, typically ranging from femtomolar to picomolar (fM-pM) levels [9]. In contrast, conventional colorimetric enzyme-linked immunosorbent assays (ELISAs) generally offer sensitivity only at the nanogram per milliliter (ng/mL) level, while more sensitive chemiluminescent ELISA methods can reach picogram per milliliter (pg/mL) levels [10]. However, even the more sensitive chemiluminescent ELISA still falls short of the fM-pM sensitivity required for early-stage biomarker detection. This technical discrepancy causes a substantial number of early pathological signals to be masked by the complex background of biological fluids, thereby hindering the accurate interpretation of peripheral signals that have crossed the blood–brain barrier [10]. Therefore, developing novel diagnostic technologies capable of overcoming sensitivity limitations while enabling non-invasive or minimally invasive detection has become a core scientific challenge that urgently needs to be addressed in the field of early and precise diagnosis of neurological diseases [11]. To simultaneously address the two major challenges of insufficient sensitivity and the need for non-invasive sampling, nanomaterial-based biosensing technologies (nanobiosensors) are regarded as one of the most promising approaches.

The essence of biosensing technology lies in the pursuit of detection sensitivity and signal transmission efficiency, as shown in Table 1. Driven by the demand for early diagnosis of neurological diseases, this technology is evolving from macroscopic optical detection toward interface-based signal amplification, specifically at the electrode-electrolyte interface (where nanomaterials accelerate charge transfer) and the analyte–biorecognition element interface (where target binding events are precisely transduced). Early immunoturbidimetry was constrained by signal thresholds, resulting in low sensitivity [36]. Although ELISA improved detection sensitivity to the nanomolar/millimolar level through enzymatic amplification, their multi-step procedures and medium limitations render them incapable of meeting the detection requirements for biomarkers in the femtomolar to picomolar (fM-pM) concentration range [10,12]. Electrochemical biosensors offer real-time responses. However, conventional planar electrodes suffer from limited specific surface area and restricted interfacial charge transfer kinetics, making it difficult to stably capture ultra-low-concentration neurological biomarkers [37,38]. The key to overcoming the sensitivity bottleneck of traditional sensors lies in the unique size effects and interfacial characteristics of nanomaterials [39,40]. Nanomaterials, characterized by high specific surface area, efficient charge transfer capability, and programmable functionalization, synergistically enhance sensitivity, selectivity, and antifouling capacity, three core attributes essential for clinical translation. Typically defined as materials with dimensions of 1–100 nm, nanomaterials possess a remarkably high surface-area-to-volume ratio that gives rise to unique physicochemical properties, including enhanced catalytic activity, tunable optical emission, and superior electrical performance [41,42]. First, nanomaterials serve as effective platforms for molecular immobilization and also as “accelerators” of charge transfer kinetics. For instance, in the development of electrochemical biosensors for detecting ultra-low concentrations of amyloid-beta oligomers in early Alzheimer’s disease diagnostics, gold nanoparticles with optimized size significantly reduce interfacial impedance by enhancing the electroactive surface area and electron conduction pathways [43,44]. This specific nanomaterial integration enables the rapid capture of femtogram-level signals that were otherwise undetectable for the target neurological biomarkers [45,46]. Second, the rich surface chemistry of nanomaterials enables tailored interfacial engineering, a capability fundamentally difficult to achieve with traditional macroscopic planar electrodes. Specifically, nanomaterials allow for the active design and precise control of the sensing microenvironment, such as optimizing the spatial orientation, adjusting the immobilization density, and preserving the active conformation of biorecognition elements (e.g., antibodies or aptamers) to maximize target capture efficiency [47]. Chemical modification strategies, such as surface ligand engineering, enhance the charge transfer rate at the electrode-solution interface, leading to faster electron transmission, shorter response times, and higher signal intensity [47]. Furthermore, by designing nanocomposites such as molecularly imprinted composite polymers (MICP), minute conductance changes induced by molecular binding can be detected, allowing the capture of biomarkers at picomolar concentrations [48,49]. In summary, the integration of nanomaterials facilitates a transition from macroscopic solution detection to microscopic interfacial regulation. This approach not only resolves the long-standing issues of insufficient sensitivity and restricted interfacial reactions associated with traditional methods but also establishes a comprehensive technical framework that spans from single-analyte enrichment to multiplexed signal output [50], thereby laying a solid foundation for addressing the challenges of early diagnosis in neurological diseases [11,51].

This review focuses on how nanomaterial interface engineering can address the trade-off between sensitivity and selectivity in biosensors, mitigate biofouling, enable multiplexed detection in complex biological fluids, and ultimately facilitate the clinical translation of early and minimally invasive neurodiagnostic technologies.

## 2. Nanomaterial Systems and Integrated Sensing Modes

The performance of nanobiosensors is fundamentally determined by their interfacial design, which has enabled the widespread use of nanomaterials in modern biosensors. This broad applicability relies on two key factors: the deep integration of nanomaterials with sensing mechanisms, and the continuous optimization of molecular recognition elements. This chapter focuses on the functional roles and sensing principles of nanomaterials in electrochemical biosensors, providing a systematic framework for understanding their fundamental basis in the diagnosis of neurological diseases.

### 2.1. The Functional Role of Nanomaterials in Biosensors

Signal amplification begins with nanomaterials themselves, which serve as efficient signal enhancers due to their high specific surface area and their geometric dimensions (1–100 nm) being comparable to those of biomolecules such as proteins and nucleic acids [52]. This size compatibility allows high-density immobilization of recognition probes and facilitates effective target capture, thereby enhancing detection sensitivity [53,54]. Complementing this effect is the optimization of the molecular recognition interface. Nanomaterials possess programmable surface chemical properties [52], and their extremely high specific surface area along with abundant surface modification sites enable precise tuning of the nanointerface chemistry via self-assembled monolayers or covalent coupling strategies [55]. Compared with conventional planar surfaces, where the limited surface area and sparse functional groups often lead to random bioreceptor orientation, low immobilization density, and significant nonspecific adsorption, nanostructured surfaces offer distinct advantages. The high curvature of certain nanomaterials (e.g., nanoparticles with diameters of 5–20 nm) favors site-specific attachment of bioreceptors via terminal groups (e.g., the Fc region of antibodies) due to reduced steric hindrance. Additionally, the high density of functional groups on nanomaterial surfaces allows precise control over immobilization density and orientation, maximizing the accessibility of active binding sites while minimizing nonspecific adsorption [56,57,58,59]. Furthermore, nanomaterials enable high-precision spatial positioning. For example, the tip of a nanoelectrode can enhance the spatiotemporal resolution of electrochemical sensing down to the single-cell level, facilitating the precise capture of signals from subcellular regions [60,61].

### 2.2. Classification of Nanobiosensors: Sensing Modalities and Material Systems

Different sensing principles impose distinct requirements on the physicochemical properties of nanomaterials. The appropriate matching between material characteristics and sensing principles is critical for achieving high-performance detection. Table 2 summarizes common types of nanomaterials and their applicable sensing principles. In the following subsections, representative nanomaterial systems are introduced according to their underlying sensing principles.

#### 2.2.1. Electrochemical Sensing: Carbon-Based Nanomaterials

**Figure 1:**
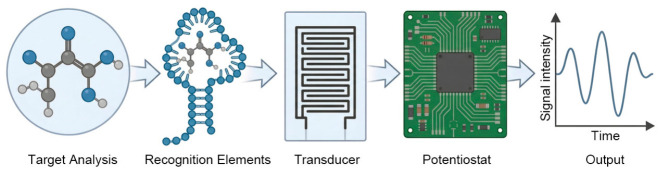
Principle of Electrochemical Biosensor.

Electrochemical biosensors achieve quantitative detection by immobilizing biorecognition elements (e.g., antibodies) onto solid-phase carriers and leveraging specific molecular interactions. In nanomaterial-enhanced biosensors, the analyte diffuses to the biorecognition layer on the sensing surface, where molecular recognition occurs. This interaction produces chemical signals that are amplified or modulated by the nanomaterials and subsequently converted into measurable electrical signals by a transducer [52,82,83] (see Figure 1). During this process, nanomaterials play multiple functional roles, which can be categorized into three main aspects: signal amplification, optimization of the molecular recognition interface, and spatial positioning.

Carbon-based nanomaterials, such as graphene and carbon nanotubes, have come to dominate the field of electrochemical biosensing due to their high conductivity, wide potential window, and excellent printability [84]. These materials enable the construction of sensing interfaces with high specific surface area and rapid electron transfer kinetics, facilitating high-sensitivity real-time monitoring of neurotransmitters (e.g., dopamine and serotonin). Consequently, they are widely integrated into implantable electrodes and brain–computer interfaces, providing valuable support for research into the mechanisms of neurological disorders such as Parkinson’s disease.

For instance, the carbon-coated microelectrodes (CCMs) developed by Qi et al. can capture millisecond-scale dynamic changes in dopamine in cerebrospinal fluid with sub-second temporal resolution, achieving a detection limit as low as 5 nM [27]. This capability significantly enhances the analysis of neurotransmitter release and reuptake processes [27]. Furthermore, composites of carbon nanotubes and graphene quantum dots exhibit excellent electrocatalytic selectivity, enabling the simultaneous detection of dopamine, uric acid, and ascorbic acid, thereby offering a new paradigm for multi-target analysis [64].

Despite these advantages, carbon-based electrodes face challenges related to signal attenuation caused by nonspecific adsorption of biomacromolecules in practical applications [63,65,85]. This issue also limits the sensitivity and selectivity of graphene field-effect transistors (GFETs) in complex biological fluids. To address this problem, researchers have proposed a modification strategy using fluorinated covalent organic framework (F-COFs) films. This approach effectively enhances the device’s resistance to biofouling while endowing it with molecular sieving functionality, thereby improving detection reliability [62].

#### 2.2.2. Optical/SERS Sensing: Noble Metal Nanomaterials

Noble metal nanomaterials, such as gold and silver, serve as the core components in surface-enhanced Raman scattering (SERS) and localized surface plasmon resonance (LSPR) sensing. Upon excitation at specific wavelengths, these materials generate localized surface plasmon resonance, creating electromagnetic “hotspots” that significantly amplify the Raman signal. SERS-based detection generally falls into two categories: label-free (direct) detection and labeled (indirect) detection.

Label-free SERS captures the intrinsic Raman fingerprint of the target analyte after its direct binding to the nanostructured surface. This approach is simple and rapid but may suffer from lower sensitivity. For example, a label-free SERS platform based on aptamer-modified mesoporous gold and gold-coated magnetic nanoparticles achieved a detection limit of 0.15 pM for Aβ oligomers in cerebrospinal fluid [29]. In contrast, labeled SERS uses Raman reporter molecules on recognition elements (e.g., antibodies, aptamers), offering higher sensitivity at the cost of increased assay complexity. For instance, a hybrid plasmonic nanoprobe assembled from gold nanorods and MXene quantum dots enabled detection of neurofilament light chain (NfL) in plasma at 17.38 pg/mL [70]. The aptamer-DNA enzyme-nanofilm system exhibited significantly higher sensitivity compared to conventional ELISA [86]. Thus, label-free SERS is suitable for rapid screening of molecules with strong Raman signals, while labeled SERS is preferred for ultra-sensitive detection of low-abundance biomarkers. Overall, the choice depends on the balance between speed/simplicity and sensitivity/multiplexing capability.

Although noble metal nanoparticles offer advantages such as high sensitivity and low cost, they remain susceptible to matrix interference when analyzing complex biological samples. Moreover, the controllability of plasmonic properties during large-scale production poses a key challenge for clinical translation. To address these issues, current strategies include the addition of antifouling layers onto gold surfaces, as well as the combination of ultraviolet laser interference lithography with ion milling. These approaches have reduced the standard deviation of the LSPR wavelength to within 10 nm, thereby significantly improving the reproducibility of the fabricated nanomaterials [59,68,69,70,71,87,88].

#### 2.2.3. Fluorescence/Photoelectrochemical Sensing: Quantum Dots and Semiconductor Materials

In the field of fluorescence sensing, materials such as quantum dots and silicon nanoparticles are widely employed due to their tunable emission spectra and high quantum yield. The detection mechanisms typically rely on fluorescence resonance energy transfer (FRET) or photoinduced electron transfer (PET) [89].

In photoelectrochemical (PEC) sensing, semiconductor materials enable detection through the efficient separation of photogenerated electron-hole pairs. For example, the Yang team utilized hydrogen bonding interactions between silicon nanoparticles and neurotransmitters to construct a synchronous fluorescence spectroscopic fingerprinting platform, which successfully distinguished dopamine, norepinephrine, and epinephrine, achieving a detection limit as low as 2.50 nM [90]. Quantum dot-based paper sensors enabled visual detection of dopamine with a detection limit as low as 0.38 nM, providing a portable solution for point-of-care testing [65].

In the PEC domain, the Zhang team developed a single-atom-site-modified Zn-N_4_/TiO_2_ sensor that employs atomic-level “molecular docking” to target norepinephrine. With a response time of only 60 ms, this sensor successfully achieved synchronous monitoring of multiple brain regions in epileptic mice [28].

Despite these advancements, single-atom materials face challenges such as reduced catalytic activity and high preparation costs [80,81]. Preventing atomic aggregation under harsh operating conditions remains a critical concern. Integrating single-atom catalysts into microdevices and establishing standardized metrics that bridge theoretical predictions with practical performance are key strategies for addressing these challenges [80,81].

#### 2.2.4. Magnetic Nanomaterials: Separation, Enrichment, and Multimodal Detection

Magnetic nanoparticles, such as Fe_3_O_4_, are primarily employed as carriers for the capture of trace target substances in complex samples. They are often integrated with techniques such as SERS and SPR to achieve multimodal and highly sensitive detection.

For instance, Fernandez Flores et al. developed an antibody-coated magnetic nanowire-based ExoAssay capable of rapidly isolating central nervous system-derived exosomes from plasma and detecting the levels of Aβ and p-tau [76]. Jang et al. utilized transferrin-modified magnetic nanoparticles to separate brain-derived exosomes from the plasma of Parkinson’s disease patients within 35 min, subsequently analyzing the expression profiles of eight microRNAs [91]. Furthermore, gold-coated magnetic nanoparticles combined with aptamer-modified mesoporous gold were used to construct a label-free SERS platform for the detection of Aβ oligomers [29].

Functionalized magnetic nanoparticles offer advantages such as high purity and high recovery rates, enabling specific capture of exosomes from complex biological matrices. Nevertheless, further improving capture efficiency and achieving simultaneous detection of multiple targets remain technical challenges that need to be addressed [78].

Beyond multimodal signal readout, magnetic micro- and nanoparticles hold a profound strategic importance in the advancement of point-of-care testing (POCT) devices, particularly for electrochemical biosensing platforms [92,93]. In clinical diagnostics, the low concentration of neurological biomarkers often necessitates significant sample pre-treatment. Magnetic supports serve as highly dispersible ‘mobile substrates’ that can rapidly capture and pre-concentrate trace targets from large volumes of complex biofluids (e.g., plasma or CSF). By applying a simple external magnetic field, the biomarker-loaded beads can be effortlessly isolated, thoroughly washed to eliminate matrix interferents, and seamlessly localized onto the surface of screen-printed electrodes. This magnetic-assisted pre-concentration not only significantly amplifies the electrochemical signal but also simplifies the analytical workflow, making it a cornerstone strategy for developing robust, field-deployable diagnostic tools.

#### 2.2.5. Electrocatalytic and Synergistic Sensing: Metal Oxides and Hybrid Nanomaterials

Beyond carbon-based and noble metal platforms, transition metal oxides (e.g., CeO_2_, MnO_2_, ZnO) and their hybrid nanocomposites have become indispensable in biosensor development due to their exceptional electrocatalytic activity, rich oxygen vacancies, and cost-effectiveness. These metal oxides offer variable valence states that significantly accelerate the redox kinetics of target analytes. However, to overcome the inherent limitation of low electrical conductivity associated with pure metal oxides, they are frequently integrated with highly conductive carbon nanomaterials or noble metals to form synergistic hybrid systems.

In these hybrid architectures, the components compensate for each other’s deficiencies: the metal oxides provide high-density catalytic active sites for biomolecular recognition, while the carbon or noble metal matrix ensures ultra-fast electron transfer pathways. For instance, hybrid interfaces combining metal oxides with graphene or carbon nanotubes have demonstrated remarkable signal amplification capabilities for detecting neuro-inflammatory biomarkers and key neurotransmitters, such as dopamine and hydrogen peroxide (H_2_O_2_) in the central nervous system [94,95]. By combining multiple transduction mechanisms, these hybrid nanomaterials successfully overcome the performance bottlenecks of single-component materials, offering a highly versatile platform for complex neurological diagnostics.

### 2.3. Design and Integration of Molecular Recognition Elements

Molecular recognition elements serve as the “sensing front-end” of biosensors, directly determining the selectivity, affinity, and overall analytical performance of the sensor. From a developmental perspective, molecular recognition elements exhibit two major evolutionary trends: the shift from biological sources to artificial synthesis, and the transition from single-mode recognition to conformational regulation and synergistic integration.

**Figure 2:**
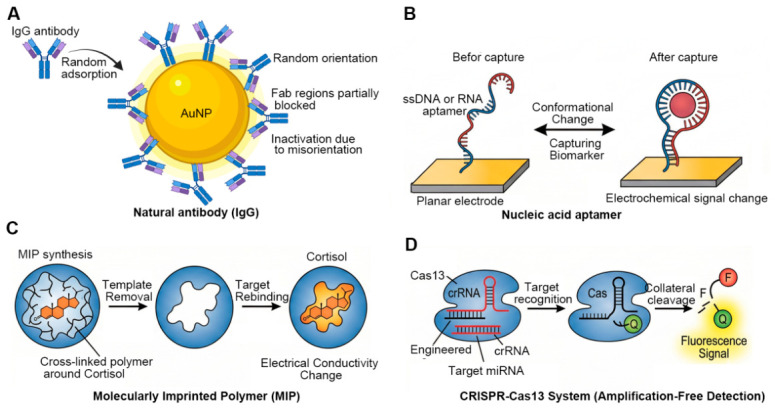
Four typical molecular recognition strategies in nanobiosensing. (**A**) Random antibody immobilization on gold nanoparticles (AuNPs) leading to reduced binding efficiency; (**B**) Target-induced conformational change in aptamers generating electrochemical signals; (**C**) Molecularly imprinted polymers (MIPs) for specific cortisol recognition; (**D**) CRISPR-Cas13-based amplification-free miRNA detection via collateral cleavage.

This subsection systematically reviews the research progress of four representative recognition elements (see Figure 2) (antibodies, nucleic acid aptamers, molecularly imprinted polymers (MIPs), and the CRISPR-Cas system) in the context of neurological disease diagnosis, with a particular focus on their synergistic integration mechanisms with nanomaterials. Among these, the CRISPR-Cas system stands out as an emerging tool for nucleic acid detection. Unlike traditional PCR, which requires complex thermal cycling and extensive sample preparation, CRISPR-Cas systems offer rapid, isothermal target recognition with collateral cleavage-based signal amplification, making them highly advantageous for decentralized testing and point-of-care applications. Furthermore, the programmability of CRISPR-Cas systems allows them to be readily integrated with nanomaterial-based signal transduction platforms, as will be discussed in the following sections.

#### 2.3.1. Antibodies: Advantages and Limitations of Classic Recognition Elements

Antibodies (Figure 2A), as classic recognition elements, offer the advantages of high specificity and high affinity. Monoclonal antibodies, in particular, have been widely employed for the immunological detection of various protein biomarkers [96,97,98]. For example, antibody-based electrochemical immunosensors have been successfully applied to the quantitative analysis of Aβ42 and phosphorylated tau protein in the cerebrospinal fluid of Alzheimer’s disease (AD) patients, achieving detection limits at the picogram per milliliter level [98,99].

However, the production of antibodies relies on animal immunization or hybridoma technology, which suffers from significant batch-to-batch variability, high costs, and limited thermal stability [100,101]. Furthermore, antibodies are prone to conformational changes and nonspecific adsorption in complex biological fluids, leading to sensor signal drift and false-positive results [100,102]. These drawbacks have driven researchers to seek more stable and controllable alternative recognition elements [11,103].

#### 2.3.2. Nucleic Acid Aptamers: Programmable Chemical Antibodies

Nucleic acid aptamers (Figure 2B) are single-stranded DNA or RNA oligonucleotides obtained through in vitro selection techniques (SELEX). They are capable of specifically recognizing proteins, small molecules, and even ions [104,105,106]. Compared with antibodies, aptamers offer several significant advantages: they can be chemically synthesized, exhibit high batch-to-batch stability, support programmable modification, and tolerate harsh conditions, thereby enabling a broader range of applications [106,107,108].

More importantly, the conformational flexibility of aptamers allows them to recognize subtle structural differences in target molecules through an induced-fit mechanism. This property is particularly crucial for the conformation-selective detection of proteins associated with neurodegenerative diseases [109,110,111].

In recent years, artificial intelligence (AI)-assisted SELEX technology has markedly accelerated the aptamer screening process. By applying machine learning algorithms to perform in-depth analysis of SELEX data, high-affinity, high-specificity candidate sequences can be rapidly identified, providing a powerful tool for the discovery of aptamers relevant to neurodegenerative diseases [41,112,113,114]. For instance, a recent study by Amu et al. employed a machine learning-powered post-SELEX modification strategy for a sclerostin-targeting aptamer. Using a stacking ensemble model (combining Random Forest, XGBoost, and LightGBM) trained on 422 modified aptamer-target affinity datasets, the model achieved a correlation coefficient of 0.82 between predicted and actual binding affinities. The AI-optimized aptamer showed 105-fold higher affinity (picomolar KD) and 3.2-fold greater bioactivity compared to the unmodified aptamer [115]. This example highlights how AI can quantitatively enhance aptamer performance, moving beyond traditional trial-and-error screening. Furthermore, the application of bioinformatics tools enables efficient aptamer selection from high-throughput sequencing data, effectively overcoming the time-consuming and computationally intensive challenges inherent in traditional screening workflows [114,116,117].

Despite their numerous advantages, it is critical to acknowledge the inherent limitations of these nucleic acid-based receptors, particularly regarding their analytical robustness under non-standardized conditions. Recent comprehensive studies have demonstrated that the biorecognition phenomenon of aptamers is highly dependent on environmental micro-conditions, most notably the ionic strength and the specific buffer composition [118,119,120]. Because aptamer target binding relies on precise three-dimensional folding dictated by intramolecular interactions, fluctuations in salt concentrations or pH in complex biological fluids can significantly alter their conformation and compromise binding affinity. Consequently, while aptamers perform exceptionally well in optimized laboratory buffers, their direct transition to analyzing raw clinical samples often requires rigorous re-validation and potential sequence re-engineering to maintain analytical robustness.

#### 2.3.3. Molecularly Imprinted Polymers: Synthetic Antibodies and Interface Engineering

MIPs (Figure 2C) are synthetic materials that achieve specific recognition by constructing three-dimensional cavities within the polymer matrix. These cavities are designed to match the shape, size, and functional group distribution of the target molecule [121,122,123]. MIPs exhibit excellent stability, resistance to high temperatures and pressures, and can be stored for extended periods without risk of biological contamination; therefore, they are often referred to as “synthetic antibodies” [121,124,125]. When combined with nanomaterials such as metal nanoparticles, carbon nanotubes, and quantum dots, MIPs can construct sensing platforms with performance comparable to traditional antibody-based sensors [89,96,123,125].

To address the challenge of distinguishing structural analogues of neurotransmitters (dopamine, norepinephrine, and epinephrine), which differ by only a single hydroxyl group, researchers employed molecular imprinting technology to create selective cavities for norepinephrine. The selectivity was provided by the MIPs, while the rapid signal acquisition (response time of 60 ms) was achieved by coupling with photoelectrochemical detection, which offers high temporal resolution [126,127]. It should be noted that such rapid response primarily reflects the detection system rather than the MIPs themselves, as the diffusion and binding of target molecules to the imprinted cavities still occur on a timescale of seconds to minutes. Nevertheless, for pre-equilibrated systems or continuous flow setups, the 60 ms response time demonstrates the sensor’s capability to track rapid concentration fluctuations once target binding is established. Thus, this combination of MIP-based selectivity and photoelectrochemical readout enables real-time dynamic monitoring of norepinephrine. For dopamine detection, researchers developed an integrated microneedle strip biomimetic sensor utilizing MIPs as the biomimetic recognition matrix. This device enables rapid, label-free detection, providing a convenient tool for point-of-care diagnosis [128]. Furthermore, a boronic acid affinity-based MIP organic electrochemical transistor (OECT) sensor was developed for dopamine detection. The OECT employed polyaniline (PANI) as the organic channel material; the specific binding of dopamine to boronic acid groups on the MIP-modified gate electrode alters the gate potential, which modulates the doping state and conductivity of the PANI channel, thereby generating a sensitive and selective electrochemical signal [129,130].

The synergistic interaction between MIPs and nanomaterials enhances sensor performance in several ways. Modifying MIPs on the surface of carbon nanotubes or graphene increases the density of imprinting sites by exploiting the high specific surface area of nanomaterials, while simultaneously lowering the detection limit through improved electron transfer efficiency [131,132]. This principle has been extended to design solid-state nanopore systems, where each nanopore functionalized with a specific MIP enables multiplexed detection, controlled release, and edge computing of neurotransmitters such as dopamine, γ-aminobutyric acid (GABA), and histamine [133,134,135]. Beyond small-molecule neurotransmitters, MIPs can also mimic antibody recognition for protein biomarkers, as demonstrated in the detection of AD-related Aβ protein, where they avoid issues of biological contamination and batch-to-batch variability [136].

#### 2.3.4. CRISPR-Cas System: A Paradigm Shift in Nucleic Acid Recognition

The introduction of the CRISPR-Cas system (Figure 2D) has brought about a paradigm shift in the detection of nucleic acid biomarkers [137,138]. When combined with nanomaterial transducers, the CRISPR-Cas12/13 system enables specific recognition of target nucleic acid sequences without the need for traditional PCR amplification, significantly shortening the turnaround time compared to traditional PCR-based methods [138,139,140]. This strategy is particularly well-suited for the direct quantitative analysis of trace (fM-level) neuron-related microRNAs in peripheral blood [30,33,138,141,142,143]. Importantly, while this rapid detection capability is essential for developing future point-of-care diagnostic devices, most current systems remain at the laboratory prototype stage.

Recent studies have substantiated the advantages of this approach. For instance, a comprehensive summary of label-free microRNA biosensing technologies based on CRISPR/Cas12a, Cas13a, and Cas14a systems highlighted the critical role of nanomaterial-assisted platforms, including gold nanoparticles, silver nanoparticles, carbon nanotubes, quantum dots, silica nanostructures, and magnetic composites, in signal enhancement. These studies underscore the significant potential of such platforms for amplification-free, high-sensitivity nucleic acid detection [144]. Consequently, the integration of the CRISPR-Cas system with nanomaterial transducers is poised to become a key direction for next-generation molecular diagnostics [137,140].

Building on these technological advances, researchers have applied CRISPR-based platforms to the diagnosis of specific neurological diseases, most notably Alzheimer’s disease (AD). In this field, a digital detection method termed “CRISPR-AD” was developed for the combined detection of proteins and microRNAs in blood, substantially improving AD diagnostic performance [142]. This platform combines the precise recognition capability of CRISPR/Cas12a with the signal amplification effect of nanomaterials to achieve highly sensitive and specific detection of AD-related biomarkers [138,140]. Guo and colleagues developed a label-free electrochemiluminescence (ECL) biosensing platform based on CRISPR/Cas12a using Aβ oligomers as target biomarkers, achieving ultrasensitive and accurate detection without additional signal amplification and providing an innovative strategy for early AD diagnosis [142]. Furthermore, researchers have combined CRISPR/Cas12a gene editing technology with nanopipettes to construct a dual-mode signal sensor for Aβ oligomer detection; the fluorescence response exhibited a strong linear relationship with AβO concentration (R^2^ = 0.99557), demonstrating the expanded applicability of CRISPR technology for protein biomarker detection [145]. Extending beyond AD, Jahani et al. reported a gold nanoparticle-assisted CRISPR-Cas12a enhanced fluorescence method for ultrasensitive detection of NfL, achieving a detection limit at the attomole level and providing a highly sensitive tool for early screening of neurodegenerative diseases [146].

The application of CRISPR-based diagnostics extends beyond AD to other neurological disorders, such as Parkinson’s disease (PD). Researchers have developed a CRISPR-based glass fiber smart bedside diagnostic platform that quantitatively detects multiple cytokines in PD patient serum. This platform employs an antibody-DNA conjugate strategy, where cytokine binding triggers the activation of CRISPR-Cas12a, leading to amplified signal readout [147]. The system also employs machine learning algorithms for intelligent data analysis, successfully distinguishing PD from atypical Parkinsonian syndromes [148]. CRISPR-Cas12a-based sensors have also been used to detect PD-related α-synuclein gene mutations and polymorphisms in dopamine metabolism-related genes [149]. Extending beyond PD, Zhou and Zhang’s team developed a CRISPR-Cas13a-based three-dimensional nanostructured surface plasmon resonance imaging sensor. By orienting recognition probes on a tetrahedral DNA scaffold and amplifying signals via CRISPR transcription-cleavage activity, they achieved amplification-free detection of AD-related miR-137 [132].

It is worth noting that the role of the CRISPR-Cas system in neurodegenerative diseases extends beyond diagnosis and demonstrates significant therapeutic potential. A systematic review comprehensively evaluated the latest advancements of this technology in this field, covering mechanism research, therapeutic applications, and translational challenges [137]. Accordingly, with continuous optimization of delivery systems, integration of artificial intelligence, and refinement of regulatory frameworks, CRISPR-Cas technology is expected to become a disease-modifying intervention for neurodegenerative diseases.

#### 2.3.5. Immobilization and Functionalization Strategies

The performance, stability, and reproducibility of a biosensor depend not only on the intrinsic affinity of the biorecognition element but also critically on how effectively it is immobilized onto the nanomaterial transducer. Improper immobilization can lead to random molecular orientation, steric hindrance, molecular leaching, and ultimately, a severe loss of bioactivity. Consequently, several functionalization strategies are strategically employed to construct stable and highly active sensing interfaces:

Covalent coupling: This remains the gold standard for achieving high long-term stability. Common strategies include EDC/NHS crosslinking chemistry (which forms stable amide bonds between carboxyl and amine groups of proteins and functionalized carbon/metal oxide nanomaterials) and thiol-metal chemistry (e.g., forming robust Au-S bonds between thiolated DNA/aptamers and noble metal nanoparticles) [150]. Covalent bonding prevents sensor degradation and allows for targeted orientation.
Affinity-based immobilization: Interactions such as the biotin-streptavidin system are widely favored due to their exceptionally high affinity and structural stability under varying pH and temperature conditions. This approach allows for the highly directional orientation of bulky recognition elements like antibodies, maximizing their capture efficiency.
Physical adsorption: While it offers the advantage of simplicity without requiring chemical modification, adsorption via electrostatic or Van der Waals interactions is prone to desorption during washing steps or in complex biological matrices, and is therefore typically used in conjunction with protective membranes.
Surface passivation and spacer engineering: To prevent steric hindrance and nonspecific fouling, nanomaterial surfaces are frequently pre-functionalized with self-assembled monolayers (SAMs) or polymer brushes (such as PEG or zwitterionic layers). These functional interfaces serve as precisely controlled scaffolds, ensuring optimal spacing between adjacent bioreceptors to maintain their native three-dimensional conformations [151,152].

By meticulously tailoring the immobilization strategy to the specific nanomaterial–bioreceptor pair, the sensing interface can effectively translate molecular recognition events into amplified measurable signals.

#### 2.3.6. Synergistic Integration of Multiple Recognition Elements and Future Prospects

The high performance of nanobiosensors stems from the deep integration of nanomaterial selection, biorecognition elements, and their integration [153]. As the understanding of molecular pathological mechanisms underlying neurological diseases continues to deepen [145], single-mode recognition strategies are no longer sufficient to meet the precise detection requirements for trace and dynamic biomarkers in complex biological samples [154,155]. Consequently, the synergistic integration of multiple recognition elements is emerging as a key strategy to enhance sensor performance [156]. For instance, combining aptamers with magnetic nanoparticles to construct a “dual-lock” recognition system significantly improves selectivity and anti-interference capability for low-abundance biomarkers [157]. In one study, specific aptamer recognition was integrated with CRISPR/Cas12a-mediated signal amplification to simultaneously detect Aβ oligomers and phosphorylated tau protein, achieving high-sensitivity, dual-target detection of AD-related protein biomarkers [158]. Furthermore, integrating the CRISPR system with aptamer sensors enables the detection of protein biomarkers via aptamers and nucleic acid biomarkers via CRISPR, thereby facilitating multimodal parallel detection on a single platform [159]. Additionally, researchers have developed a spatiotemporally hierarchical CRISPR cascade system based on DNA tetrahedral splitting, combined with gold-platinum nanolabels and artificial intelligence technology, to achieve ultrasensitive, one-step, single-tube rapid detection of miRNA-21 with a detection limit as low as 38 aM [160]. Although this study focused primarily on cancer, its design concept provides an important reference for multiplex biomarker detection in neurological diseases [156].

Artificial intelligence is accelerating the development of recognition elements. AI techniques have been applied to optimize aptamer screening conditions (e.g., GRAPE-LM achieves one-round evolution with superior affinity vs. multi-round SELEX [161]), predict optimal functional monomer combinations for MIPs (e.g., computational modeling achieves ΔEC up to −135 kcal·mol^−1^ [162]; ML models achieve R^2^ = 0.937 for imprinting factor prediction [163]), and design specific CRISPR guide RNAs (e.g., CGD outperforms existing methods via Elastic Net regression [164]). These AI-driven approaches have significantly improved development efficiency and recognition element performance [114,165,166]. For example, the CRISPRdx platform employs an AI-driven CRISPR-Cas12a multiplex detection system to identify neurogenetic markers in minimally invasive body fluids, offering a rapid and cost-effective solution for bedside pediatric genetic diagnosis [167]. With the deep integration of synthetic biology, nanotechnology, and information science, programmable, multifunctional, and intelligently responsive biosensing systems are expected to provide key technical support for achieving early and precise diagnosis of neurological diseases [155,156]. The rational design of nanomaterial interfaces and molecular recognition elements lays the foundation for translating nanobiosensors from laboratory prototypes into clinical diagnostic tools. The following sections will focus on their practical performance in quantifying neurological biomarkers and addressing the bottlenecks of clinical translation.

#### 2.3.7. Biofouling Mitigation and Interfacial Shielding Strategies

**Figure 3:**
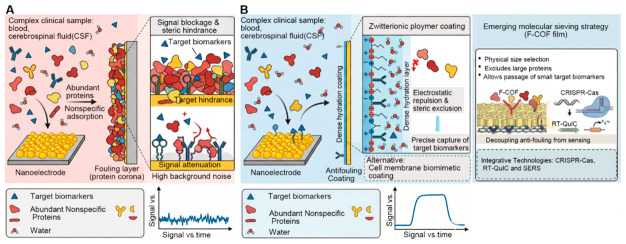
Antifouling mechanism of nanoelectrode biosensors for clinical sample detection. (**A**) Unmodified nanoelectrodes suffer from nonspecific protein adsorption (protein corona) in complex clinical samples (blood, CSF), resulting in signal blockage, high background noise, and target binding hindrance. (**B**) Anti-pollution interface engineering strategies include: hydrophilic coatings such as zwitterionic polymers and biomimetic cell membranes, and physical sieving methods such as fluorinated covalent organic frameworks (F-COFs) and molecular sieving.

Protein nonspecific adsorption (biofouling) has long been a core obstacle limiting the application of nanobiosensors in complex physiological environments such as cerebrospinal fluid and blood [168,169,170]. Biofouling not only passivates the sensor interface and impedes electron transfer but also causes signal drift and loss of sensitivity and selectivity, severely compromising the long-term stability and reliability of sensors in clinical practice [168,171]. To mitigate these issues, researchers have developed various antifouling interface design strategies that enable sensors to resist nonspecific adsorption, thereby improving performance in real biological media [62,172,173,174,175], as shown in Figure 3.

Zwitterionic polymers have become the mainstream choice for antifouling interfaces due to their unique hydration layer shielding mechanism [176,177,178]. In these materials, individual monomers contain balanced positive and negative charges, achieving overall charge neutrality and forming a dense hydration layer through strong electrostatic interactions with water molecules (see Figure 3A,B). Recent research focuses include: constructing brush-like structures on nanoporous electrodes, precisely regulating zwitterionic peptide sequences, and developing conductive antifouling composites by combining zwitterionic polymers with conjugated polymers or laser-induced graphene, all of which inhibit biofouling while maintaining electron transfer [35,179,180,181,182,183,184]. However, it should be noted that poorly designed zwitterionic coatings may introduce insulating effects and reduce signal output.

Cell membrane biomimetic coatings represent another highly promising antifouling strategy, inspired by the inherent ability of natural cell surfaces to resist nonspecific protein adsorption and immune recognition [181,185,186]. Red blood cell membranes, owing to their superhydrophilicity, low membrane protein content, and excellent biocompatibility, are the most widely used materials for antifouling modification in complex biological fluids [187]. In the field of neurological diagnosis, neuron membrane biomimetic coatings have garnered increasing attention due to their high compatibility with the central nervous system [188,189,190]. Such biomimetic interfaces maintain low fouling adhesion even in whole blood, serum, and urine, thereby supporting the development of multimodal clinical detection platforms [191,192,193,194,195].

F-COFs and molecular sieving strategies (see Figure 3A,B). Beyond zwitterionic polymers and cell membrane biomimetic coatings, molecular sieving-based antifouling strategies have emerged as another important direction in this field [196,197]. Sun and colleagues reported a fluorinated covalent organic framework (F-COF) film-modified graphene field-effect transistor biosensor, in which the F-COF film was transferred onto the channel of the graphene field-effect transistor (FET) [62]. This strategy decouples antifouling properties from molecular recognition at the level, providing a general approach for selective, real-time graphene FET biosensing in complex biological fluids [198].

In summary, current antifouling interface design has evolved from single-mechanism hydration layer shielding toward multi-mechanism synergy and structural–functional integration [196,199]. Zwitterionic polymer strategies center on controllable hydration layers, achieving performance optimization through structural engineering [198,200]. Cell membrane biomimetic strategies leverage the “camouflage” properties of natural cell membranes, demonstrating significant advantages in biocompatibility and multifunctional integration [201,202]. Molecular sieving strategies, which are based on physical size selection, offer complementary solutions for specific scenarios [203,204]. The continuous development and integration of these strategies have laid a solid interfacial chemistry foundation for noninvasive or minimally invasive detection of neurological disease biomarkers in body fluids using nanobiosensors [205].

## 3. Application of Nanobiosensors in the Diagnosis of Neurological Diseases

The most significant achievement in translating nanobiosensing technology into clinical practice is the precise detection of biomarkers associated with neurodegenerative diseases and neuropsychiatric disorders. This section evaluates the performance of nanomaterial-mediated platforms for quantitative trace analysis in complex biological systems, focusing on their applications in early screening, disease progression monitoring, and the exploration of novel biomarkers.

### 3.1. For Early Screening: High-Sensitivity Quantitative Detection of Established Protein Biomarkers

**Figure 4:**
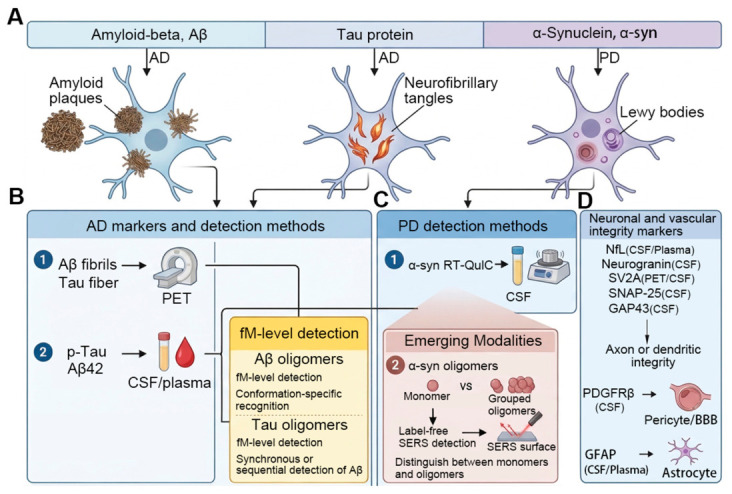
Biomarkers for neurodegenerative diseases. (**A**) Biomarkers for AD and PD; (**B**) Detection of AD-related proteins; (**C**) Detection of PD-related proteins; (**D**) Biomarkers related to the integrity of neurons and blood vessels.

Misfolded protein aggregates, primarily amyloid-beta (Aβ), phosphorylated tau protein (p-tau), and α-synuclein, are critical for the differential diagnosis of AD and PD [206,207]. Nanomaterial-enhanced electrochemical and optical detection methods are progressively replacing traditional neuroimaging techniques, achieving sub-picomolar detection sensitivity (see Figure 4).

The detection of AD-related proteins (Aβ and p-tau) is shown in Figure 4A,B. Leveraging the high specific surface area of gold nanostars and their specific recognition capability for DNA aptamers, researchers have developed a graphene-based field-effect transistor biosensor that enables quantitative detection of Aβ1-42 in undiluted plasma, with a detection limit as low as 1.6 fM. Nanobiosensor-based platforms have demonstrated superior diagnostic performance for AD compared to traditional plasma ELISA (typically AUC ≈ 0.78), with multiple studies reporting AUC values above 0.90 and sensitivities/specificities exceeding 90% [208]. The performance of this detection method is comparable to that of CSF biomarker analysis, offering a minimally invasive approach for large-scale population screening [209]. Nevertheless, this technology still faces several challenges: a lack of prospective validation in asymptomatic populations, and the fact that sensor repeatability across different production batches has not yet been systematically evaluated.

The detection of PD-related proteins (α-synuclein) is shown in Figure 4A,C. The aggregation of α-synuclein is a hallmark of Parkinson’s disease. SERS technology has enabled label-free detection of α-synuclein oligomers, a key biomarker for Parkinson’s disease [210]. Recent studies have demonstrated the potential of SERS-based platforms for PD diagnosis using biofluids such as saliva, with artificial intelligence-assisted analysis achieving high diagnostic accuracy [211]. However, this study has limitations, including a small sample size and the absence of independent validation. Furthermore, the invasive nature of CSF collection restricts its application in routine screening, while attempts to apply this method to blood or saliva have been hindered by the extremely low abundance of α-synuclein oligomers in peripheral body fluids [212]. Although the electromagnetic “hotspots” generated at the nanocarbon–metal interface significantly enhance the Raman signal, enabling differentiation between protein monomers and oligomers, most studies remain at the level of case–control cohorts with limited sample sizes (*n* < 100). Prospective validation in presymptomatic individuals and standardization of sensor manufacturing are urgent prerequisites for clinical application.

### 3.2. For Disease Progression Monitoring: Real-Time Monitoring of Dynamic Neurochemical Substances

Unlike the relatively static protein markers associated with neurodegenerative diseases, neuropsychiatric disorders, such as major depressive disorder and schizophrenia, require tracking highly dynamic fluctuations in neurochemical substances.

Neurotransmitter array detection. Imbalances in neurotransmitters, including dopamine, serotonin, and glutamate, represent a key pathological mechanism of depression [213]. Nanomaterial-based microelectrodes, particularly those modified with MXenes or nitrogen-doped graphene, enable sub-second resolution monitoring of dopamine and serotonin in vivo. For example, carbon-coated microelectrodes (CCMs) implanted in the nucleus accumbens of freely moving rats achieve a detection limit of 5 nM and maintain stable performance for up to four weeks, allowing longitudinal tracking of dopamine fluctuations induced by chronic fluoxetine treatment [27]. Although such tools have been extensively validated in rodent models of depression, their application in human patients remains in its early stages. Major obstacles include the invasive nature of intracranial implantation, the risk of chronic inflammatory responses (gliosis), and the difficulty of calibrating sensor signals within the complex human brain microenvironment. Recent research has focused on developing flexible, minimally invasive probes and employing surface modification with antifouling polymers to extend device lifespan; however, clinical-grade devices have yet to receive regulatory approval [214,215,216].

Stress biomarker (cortisol) detection. Dysregulation of the hypothalamic–pituitary–adrenal (HPA) axis, reflected by abnormal cortisol dynamics, is strongly associated with neuropsychiatric conditions such as post-traumatic stress disorder (PTSD), major depressive disorder, and chronic anxiety. Noninvasive detection of cortisol from sweat or saliva using MIPs combined with conductive nanostructures has opened new avenues for point-of-care diagnosis of these mental disorders (see Figure 2C). Unlike neurodegenerative disease biomarkers that require fM-pM sensitivity due to their trace concentrations in blood, cortisol circulates at nanomolar levels (normal range: 1–15 nM). Therefore, the key challenge for neuropsychiatric diagnostics is not achieving ultra-low detection limits, but rather enabling continuous, real-time dynamic monitoring in non-invasive fluids (e.g., sweat) to map HPA-axis dysfunction and capture stress-induced fluctuations. One study reported a wearable electrochemical sensor based on gold nanoparticle-doped MIPs that measures cortisol in saliva, with a detection range of 0.5–200 nM. In 40 healthy volunteers undergoing the Trier Social Stress Test, the sensor achieved a correlation coefficient of 0.94 compared with the gold-standard LC-MS/MS method [217]. These findings collectively demonstrate the strong potential of MIP-based electrochemical sensors for real-time, non-invasive cortisol monitoring in mental health applications. Despite these encouraging results, several obstacles remain: (i) sensor performance declines after 5–7 days of continuous use due to biofouling; (ii) inter-individual variations in sweat pH and ionic strength affect signal stability; and (iii) a scalable manufacturing process that ensures reproducible MIP film thickness has not yet been validated. Before these sensors can receive regulatory approval for home-based mental health monitoring, future work must address these challenges [218,219].

### 3.3. Ultrasensitive Detection of Established and Emerging Biomarkers: Exosomes and Circulating microRNAs

**Figure 5:**
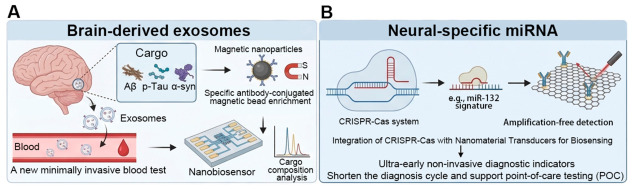
Emerging classes of biomarkers for neurological diseases. (**A**) Exosome-based biomarker analysis; (**B**) Circulating microRNA analysis.

Beyond proteins and small molecules, the diagnostic landscape has expanded to include brain-derived exosomes and circulating miRNAs, which have emerged as promising non-invasive or minimally invasive biomarkers for neurological diseases, as shown in Figure 5.

Exosome analysis (Figure 5A). Nanomaterials play a dual role in exosome analysis: they act as separators for exosome enrichment and as signal transducers for molecular profiling. Magnetic nanoparticles functionalized with specific antibodies (e.g., against CD63 or L1CAM) can rapidly enrich brain-derived exosomes from blood, followed by detection of payload proteins (such as T-Tau, P-Tau, and α-synuclein) via Raman scattering. For instance, multiple antibody-coated gold nanoparticles have been developed for rapid isolation of CNS-specific exosomes from blood, demonstrating the feasibility of nanomaterial-assisted exosome enrichment [76]. Compared with CSF biomarker analysis, exosome detection is less invasive (requiring only a blood draw) while offering comparable diagnostic accuracy (CSF P-Tau181 AUC = 0.94) [220]. However, the exosome isolation process remains cumbersome and lacks standardization, with recovery rates ranging from 30% to 70% depending on antibody batch and nanoparticle coating. Furthermore, most studies are retrospective and single-center; there is an urgent need for prospective multicenter validation to establish reference ranges and cutoff values.

Amplification-free miRNA detection (Figure 5B). Researchers have harnessed the programmable cleavage activity of CRISPR-Cas12a on two-dimensional nanomaterial surfaces (e.g., graphene or gold nanoparticles) to develop amplification-free detection methods for neuron-specific miRNAs, such as miR-132 and miR-137 [32]. In a study of 40 serum samples (20 from AD patients and 20 from controls), a CRISPR-based three-dimensional nanostructured surface plasmon resonance imaging biosensor detected miR-137 with a detection limit of 212 fM, a dynamic range spanning five orders of magnitude, and an AUC of 0.88 for AD diagnosis [132]. This method requires only 10 μL of serum sample and yields results within 45 min, whereas RT-qPCR takes 3–4 h. Moreover, CRISPR sensors exhibit excellent specificity, capable of discriminating single-nucleotide differences in miRNA sequences.

## 4. Conclusions and Future Perspectives

### 4.1. Summary and Outlook

The integration of nanomaterials into biosensing architectures has significantly advanced the transformation of early diagnosis and monitoring paradigms for neurological diseases. By leveraging the unique physicochemical properties of zero-dimensional to two-dimensional nanostructures, researchers have achieved unprecedented analytical performance, pushing detection limits to the fM and even single-molecule levels. However, despite these breakthroughs at the laboratory scale, the transition from “bench to bedside” remains hindered by several key obstacles [32].

#### 4.1.1. Balancing Sensitivity and Selectivity

In the design of nanobiosensors, balancing sensitivity and selectivity presents a key technical challenge. While pushing the limit of detection (LOD) down to the femtomolar (fM) or even attomolar (aM) range typically necessitates sophisticated interface modification and robust signal amplification strategies, such measures may compromise the sensor’s selectivity, particularly in complex biological matrices (e.g., blood or cerebrospinal fluid). In these environments, the amplification of non-specific signals readily leads to false-positive outcomes [221].

Effective strategies to address this challenge include: optimizing the spatial accessibility of recognition sites by tailoring the size and morphology of nanomaterials [222]; synergistically integrating highly specific recognition elements (e.g., aptamers, molecularly imprinted polymers) with nanomaterials; or employing dual-modal/ratio-based detection strategies that leverage internal signal calibration to eliminate background interference [181]. Future research should further reduce cross-reactivity with non-target molecules while maintaining single-molecule-level sensitivity, which remains one of the core challenges in this field.

#### 4.1.2. Multiplex Detection in Complex Matrices

In clinical practice, reliance on a single biomarker is often insufficient for the accurate diagnosis of complex neurological disorders such as Alzheimer’s disease and Parkinson’s disease, as their pathological mechanisms involve perturbations across multiple molecular pathways. Multiplex detection, the simultaneous quantification of multiple analytes from a single sample, has therefore emerged as a key advancement for improving diagnostic accuracy and enabling disease subtype stratification [223]. Thus, simultaneous detection of multiple targets (e.g., a panel comprising Aβ42, p-tau, and neurofilament light chain, or an array of neurotransmitters including dopamine, serotonin, and glutamate) holds significant value for enhancing diagnostic accuracy and enabling disease subtype stratification [223].

Nonetheless, multiplex detection confronts several practical hurdles: potential cross-interference between recognition elements for distinct targets; signal crosstalk that undermines quantitative accuracy; and technical challenges in optimizing the immobilization density and orientation of multiple capture probes on a single sensing interface [224]. To date, researchers have proposed solutions including spatially resolved multi-electrode arrays, encoding strategies based on orthogonal signal outputs (e.g., wavelength-distinct fluorescence or potential-specific electrochemical signals), and signal deconvolution via machine learning algorithms [225,226]. In the future, developing scalable, repeatable and easy-to-operate multiple detection platforms to meet the needs of high-throughput screening in clinical settings remains an important direction in this field.

### 4.2. Next-Generation Diagnostic Paradigm: Multimodal Fusion and Chemical Brain–Computer Interfaces (cBCIs)

**Figure 6:**
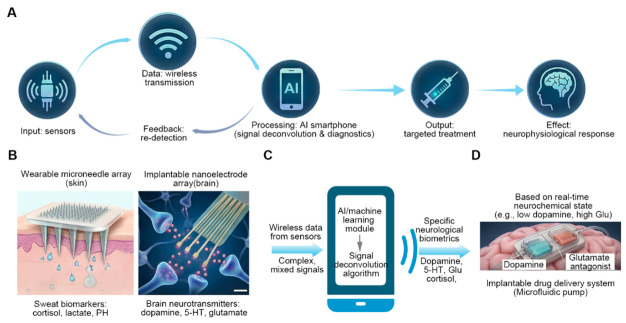
Closed-loop neurodiagnostic and therapeutic system integrating nanosensors, artificial intelligence (AI), and targeted drug delivery. (**A**) Workflow of sensing, wireless transmission, AI processing, targeted treatment, and feedback re-detection; (**B**) Wearable microneedle and implantable nanoelectrode arrays; (**C**) AI-driven signal deconvolution; (**D**) Implantable microfluidic drug delivery system for real-time neuroregulation.

Given the high heterogeneity of neuropathology, relying solely on a single biomarker or a single detection method is no longer sufficient to meet the requirements of precise diagnosis. The future of neurological diagnosis lies in the construction of a high-dimensional “spatiotemporal perception matrix” (Figure 6). Future development directions include, but are not limited to, the following three aspects.

#### 4.2.1. Deep Integration of Multimodal and Multiplexing Platforms

Next-generation diagnostic devices will no longer be limited to single-dimensional signal output; instead, they will integrate multiple sensing modalities into a single microdevice. Here, we define “deep integration” as the simultaneous, on-chip fusion of different transduction mechanisms (e.g., combined opto-electrochemical interfaces) at the device level, where the sensing modalities share a common set of electrodes, nanomaterials, or microfluidic channels to achieve synergistic signal readout. This is distinct from simple integration, which merely places two separate sensors (e.g., an electrochemical electrode and a SERS substrate) on the same physical support without functional interdependence between the modalities. For example, they can achieve simultaneous in situ electrochemical and SERS detection within the same device [227]. The cross-validation of physical and chemical signals, combined with structural neuroimaging features, will provide multidimensional panoramic diagnostic data, thereby eliminating false-positive blind spots inherent to single-technology approaches [228]. The current priority is to integrate electrochemical and SERS modalities onto a single flexible probe and validate its performance in animal models. Early proof-of-concept studies have demonstrated feasibility: for instance, a shared nanostructured gold electrode has been shown to support both electrochemical and SERS detection on the same chip, achieving simultaneous readout of dopamine and its oxidation products [229]. This suggests that deep integration is technically achievable, though extensive preclinical validation remains pending.

#### 4.2.2. Closed-Loop Chemical Brain–Computer Interfaces (cBCIs)

Another major transformation in the next-generation diagnostic paradigm is reflected in closed-loop systems that combine real-time neurochemical monitoring with on-demand therapeutic intervention (Figure 6A). Currently, wearable sensors based on flexible nanostructures can detect stress markers such as cortisol through noninvasive biofluids (e.g., sweat), enabling personalized remote patient management (Figure 6B). However, a more disruptive frontier lies in the development of “closed-loop brain–computer/chemical sensor fusion systems,” which integrate electrophysiological recordings (e.g., micro-electroencephalography) with real-time neurochemical sensing. Recent advances in multimodal on-chip sensing platforms, such as a nano-corrugated graphene device that integrates FET, electrochemical, and SERS detection on a single chip [230], provide a technological foundation for realizing such closed-loop systems.

For implantable sensors designed for real-time brain monitoring, the chronic neuroinflammatory response triggered by synthetic nanomaterials remains a primary concern. By adopting extreme biocompatibility designs to minimize immunogenicity, these devices will be capable of capturing dopamine or serotonin fluctuations at the millisecond level while releasing neuromodulatory signals or targeted drugs (Figure 6B–D), thereby achieving a seamless diagnostic–therapeutic loop. Closed-loop systems impose stringent requirements on the long-term stability of the sensing interface. Recent progress has partially addressed this challenge: for example, zwitterionic polymer coatings have extended sensor stability in vivo from days to over 4 weeks in animal models, while cell membrane-coated nanoparticles have demonstrated reduced immunogenicity and improved biocompatibility [231]. Surface engineering using zwitterionic polymers or cell membrane coatings represents a promising short-term solution, though long-term human validation is still needed.

#### 4.2.3. AI-Driven Predictive Diagnosis

Advanced machine learning and deep learning algorithms can extract complex neurochemical signals from noisy biological backgrounds. The extreme complexity of neural signals and the presence of multi-analyte interference necessitate advanced data processing. The deep integration of nanobiosensor arrays with AI-driven pattern recognition will usher in a new era of “predictive diagnosis”. AI algorithms will distill specific “neurochemical signatures” from massive, noisy multiplexed datasets, enabling the earliest possible disease risk assessment before irreversible neuronal damage occurs. Initial successes have been reported: for instance, deep learning models integrating electrochemical sensor data with clinical features have achieved AUC values above 0.85 for predicting AD progression, outperforming single-modality approaches [232]. These proof-of-concept results highlight the potential of AI-driven diagnostics, though prospective clinical validation is still required.

Although nanomaterial-based biosensors have laid a solid foundation for next-generation neurological diagnostics, overcoming numerous obstacles, including clinical matrix interference and manufacturing scalability, remains the ultimate challenge. Sustained interdisciplinary collaboration among materials scientists, neurobiologists, data scientists, and clinicians will be key to fully realizing the potential of these cutting-edge technologies and, ultimately, reversing the trajectory of neurological diseases.

## Figures and Tables

**Table 1:** Comparison of traditional diagnostic techniques and electrochemical nanobiosensing technologies.

Comparison Dimension	ELISA	MRI/PET	Mass Spectrometry Analysis	Nano-Electrochemical Sensor
Principle	ELISA based on chromogenic reaction	Magnetic resonance imaging/Positron emission tomography	Ion separation and detection based on mass-to-charge ratio	Nano-interface electrochemical signal transduction (current/impedance)
Recognition Mechanism	Antigen–antibody specific recognition	Molecular probe/tracer targeting	*m*/*z* discrimination + fragmentation pattern matching	Biorecognition elements + nanomaterial signal amplification
Invasiveness	Moderate-High (blood, CSF, or tissue sample required)	Non-invasive (MRI)/Low-Moderate (PET requires tracer injection)	Moderate-High (biopsy, CSF or blood sample required)	Low to non-invasive (adaptable to sweat/saliva/urine)
Limit of Detection (LOD)	Chemiluminescence method: pM-fM (~pg/mL)	mM-μM (MRI)/pM (PET)	fM~aM	fM~aM
Temporal Resolution	Static/hour-level (~1–4 h)	Static/minute-level (~15–60 min)	Static/minute- to hour-level	Millisecond To second-level (real-time/dynamic)
Sample Preparation	Complex (blocking, washing, incubation)	Simple (MRI)/Required (PET tracer labeling)	Complex (extraction, derivatization)	Simple (direct detection)
Multiplex Detection Capability	Low (predominantly single-analyte)	Low (MRI mainly anatomical; PET multiplexing possible but complex)	High (simultaneous multi-omics detection)	Moderate-High (array/multi-channel design)
Key Advantages	Mature, standardized, widely available instrumentation	High spatial resolution (MRI); high sensitivity (PET)	High sensitivity, high specificity, multi-omics capability	Real-time, miniaturized, low-cost, POCT-friendly
Key Limitations	Time-consuming, labeling required, poor dynamic detection	Expensive, non-portable, no real-time molecular dynamics	Expensive, complex, not suitable for point-of-care testing	Stability, consistency, interference from complex matrices
Ref.	[12,13,14,15,16,17]	[18,19,20,21,22]	[23,24,25,26]	[27,28,29,30,31,32,33,34,35]

**Non-invasive:** urine, saliva, exhaled breath, sweat. **Low-invasive:** Capillary (finger prick) blood and microdialysate samples. **Moderate-high-invasive:** venipuncture, tissue biopsy, CSF, radiotracer injection.

**Table 2:** Comparison of typical nanomaterial properties and their applicable sensing principles.

Nanomaterial Type	Carbon-Based Materials (Graphene, Carbon Nanotubes)	Precious Metal Nanoparticles (Gold, Silver)	Semiconductor Materials (TiO_2_, Quantum Dots)	Magnetic Nanoparticles (Fe_3_O_4_)	Single-Atom Material (Cu/TiO_2_)
Key properties	High conductivity, wide potential window, and suitable for screen printing	Local surface plasmon resonance (LSPR), surface-enhanced Raman scattering (SERS) activity	Photo-generated electron-hole pairs and fluorescence characteristics	Superparamagnetic, easily separable and enriched	Atomic-level dispersion sites and maximum atomic utilization rate
Electrical conductivity	Excellent	Good	Tunable	Poor	Tunable
Optical activity	None	Strong	Strong	None	None
Main applicable sensing principles	Electrochemical sensing	SERS, SPR/LSPR	Photoelectrochemistry, Fluorescence Sensing	Electrochemistry (as a carrier)	Photoelectrochemistry, Electrochemistry
Target neural markers	Dopamine (DA), norepinephrine (NE), serotonin (5-HT)	Aβ oligomers, neurofilament light chain (NfL), microRNA (miRNA)	Neurotransmitters (NE, DA), tetracycline (model drug)	Brain-derived exosomes, Aβ42, alpha-synuclein (α-syn)	Tetracycline (in vivo drug monitoring), DA
Potential clinical application (research prototype)	Real-time electrochemical monitoring of neurotransmitters	SERS/SPR detection of AD biomarkers	Fluorescence sensing of neurotransmitters, bedside testing and point-of-care diagnostics	Liquid biopsy of neurodegenerative diseases (AD, PD), isolation of brain-derived exosomes	In vivo dynamic monitoring of neurotransmitters
Key advantage	Scalable fabrication, integration with neural electrodes	Clinical cohort validation, low-cost POCT	Wearable and implantable platform compatibility Liquid biopsy	Multiplexed biomarker detection	No biological components, long-term stability
Key Limitations	Electrode fouling and biofouling, Long-term in vivo stability, Non-specific adsorption	Matrix interference, non-specific adsorption, chiral molecule detection, fabrication reproducibility	Fluorescence blinking effect, surface chemical complexity, toxicity, long-term photostability	Exosome isolation efficiency and specificity, functionalization stability, biocompatibility	Atomic agglomeration, fabrication complexity, limited enzyme activity, cost and scalability
Ref.	[27,40,62,63,64,65,66]	[29,59,67,68,69,70,71]	[72,73,74,75]	[29,76,77,78,79]	[28,30,33,80,81]

Note: The listed properties are typical, not exclusive. For instance, gold/silver nanoparticles can also be used in electrochemical sensors, and carbon nanomaterials as optical labels.

## Data Availability

Data sharing is not applicable to this article as no new data were created or analyzed in this study.

## Abbreviations

The following abbreviations are used in this manuscript:

cBCIs	closed-loop chemical brain–computer interfaces
AD	Alzheimer’s disease
PD	Parkinson’s disease
ELISA	Enzyme-linked immunosorbent assay
MRI	Magnetic Resonance Imaging
PET	Positron Emission Tomography
CSF	Cerebrospinal fluid
fM	Femtomolar
pM	Picomolar
aM	Attomolar
POCT	Point-of-Care Testing
MICP	Molecularly imprinted composite polymers
LSPR	Local surface plasmon resonance
SERS	Surface-enhanced Raman scattering
DA	Dopamine
NE	Norepinephrine
5-HT	Serotonin
NfL	Neurofilament light chain
miRNA	MicroRNA
α-syn	Alpha-synuclein
CCMs	Carbon-coated microelectrodes
GFETs	Graphene field-effect transistors
F-COFs	Fluorinated covalent organic framework
PEC	Photoelectrochemical
FRET	Fluorescence resonance energy transfer
MIPs	Molecularly imprinted polymers
SELEX	Systematic evolution of ligands by exponential enrichment
AI	Artificial intelligence
OECT	Organic electrochemical transistor
GABA	γ-aminobutyric acid
ECL	Electrochemiluminescence
Aβ	Amyloid-beta
p-tau	Phosphorylated tau protein
AUC	Area under the curve
MCI	Mild cognitive impairment
FET	Field-effect transistor
LOD	The limit of detection
